# BitPAl: a bit-parallel, general integer-scoring sequence alignment algorithm

**DOI:** 10.1093/bioinformatics/btu507

**Published:** 2014-07-29

**Authors:** Joshua Loving, Yozen Hernandez, Gary Benson

**Affiliations:** ^1^Laboratory for Biocomputing and Informatics, ^2^Graduate Program in Bioinformatics, and ^3^Department of Computer Science, Boston University, Boston, MA 02215, USA

## Abstract

**Motivation:** Mapping of high-throughput sequencing data and other bulk sequence comparison applications have motivated a search for high-efficiency sequence alignment algorithms. The bit-parallel approach represents individual cells in an alignment scoring matrix as bits in computer words and emulates the calculation of scores by a series of logic operations composed of AND, OR, XOR, complement, shift and addition. Bit-parallelism has been successfully applied to the longest common subsequence (LCS) and edit-distance problems, producing fast algorithms in practice.

**Results:** We have developed BitPAl, a bit-parallel algorithm for general, integer-scoring global alignment. Integer-scoring schemes assign integer weights for match, mismatch and insertion/deletion. The BitPAl method uses structural properties in the relationship between adjacent scores in the scoring matrix to construct classes of efficient algorithms, each designed for a particular set of weights. In timed tests, we show that BitPAl runs 7–25 times faster than a standard iterative algorithm.

**Availability and implementation:** Source code is freely available for download at http://lobstah.bu.edu/BitPAl/BitPAl.html. BitPAl is implemented in C and runs on all major operating systems.

**Contact**: jloving@bu.edu or yhernand@bu.edu or gbenson@bu.edu

**Supplementary information**: Supplementary data are available at *Bioinformatics* online.

## 1 INTRODUCTION

Sequence alignment algorithms are critical tools in the analysis of biological sequence data including DNA, RNA and protein sequences. The demands placed on computational resources by high-throughput experiments require new, more efficient methodologies. While the standard algorithms of Smith and Waterman (1981) and Needleman and Wunch (1970) calculate the score in each cell of the alignment scoring matrix sequentially, a newer technique called bit-parallelism partially overcomes score dependencies so that scores can be calculated in parallel to achieve much higher efficiencies.

Bit-parallel algorithms have been developed for exact and approximate string matching problems. Early examples include the algorithms of Baeza-Yates and Gonnet (1992), which finds exact matches to a simple string pattern, and Wu and Manber (1992), which finds approximate matches to a string pattern or a regular expression, where the number of differences between the pattern and the text is at most *k* (counting single character substitutions and single character insertions and deletions or indels). The latter is implemented as the Unix command *agrep*. Additional *k*-differences examples include (Wu *et al.*, 1996), which finds matches to ‘limited expressions’, i.e. regular expressions without Kleene closure, (Myers, 1999), which finds matches to simple string patterns and emulates the dynamic programming solution used in alignment, and (Navarro, 2004), which allows arbitrary integer weights for substitution of each pair of characters, insertion of each character and deletion of each character, and finds occurrences of regular expressions where the *sum* of the edit weights is at most *k*. In most *k*-differences algorithms, the complexity (and computing time) increases with increasing *k*.

Bit-parallel methods have been successfully applied to the longest common subsequence (LCS) problem (Allison and Dix, 1986; Crochemore *et al.*, 2001; Hyyrö, 2004), and to unit-cost edit-distance (Hyyrö and Navarro, 2005; Hyyrö *et al.*, 2005) by modifications of Myers’s method (1999). These algorithms compute the alignment score, de-linking that computation from the traceback, which produces the final alignment. In the LCS scoring matrix, scores are monotonically non-decreasing in the rows and columns, and bit-parallel implementations use bits to represent the cells where an increase occurs. In edit-distance scoring, adjacent scores can differ by at most one, and the binary representation stores the locations of (two of the three) possible differences, +1, −1 and zero. These algorithms are *ad hoc* in their approach, relying on specific properties of the underlying problems, making it difficult to directly adapt them to other alignment scoring schemes.

Below, we present a bit-parallel method for similarity and distance based global alignment using general integer-scoring (Benson *et al.*, 2013), allowing arbitrary integer weights for match, mismatch and indel. Other approaches have been suggested by Wu and Manber (1992) and Bergeron and Hamel (2002). The method of Navarro (2004) is more flexible in scoring and applies to both simple patterns and regular expressions, but is much slower than our method in practice. Our contribution is based on an observation of the regularity in the relationship between adjacent scores in the scoring matrix (Section 2.1) and the design of an efficient series of bit operations to exploit that regularity (Section 3). Because every distinct choice of weights requires a different program, we show how to construct a class of efficient algorithms, each designed for a particular set of weights, and provide an online C code generator for users. The complexity of our algorithms depends on the weights, not the ultimate score of the alignment. Our method works for general alphabets, but our interest derives from frequent use of DNA alignment when analyzing high-throughput sequencing data to detect genetic variation.

## 2 METHODS

The problem to be solved is stated in terms of similarity scoring, but the technique applies to distance scoring as well.

**Problem**

Given two sequences X and Y, of length n and m respectively, and a similarity scoring function S defined by three integer weights M (match), I (mismatch) and G (indel or gap), calculate the global alignment similarity score for X and Y using logic and addition operations on computer words of length w.

We are interested in two measures of efficiency for the algorithms. The first is standard time complexity and the second is a ratio of the word size, *w*, and the count, *p*, of logic and addition operations required to process *w* consecutive cells in the alignment scoring matrix. The efficiency, e=w/p, is the average number of cells computed per operation. For example, when using 64 bit words, LCS has e=64/4=16 [*P* = 4 operations per word (Hyyrö, 2004)], and edit distance has e=64/15≈4.2 [an improvement from 64/16 in the method of Hyyrö *et al.* (2005) and Myers (1999); see Supplementary Information for details]. As *P* is independent of *w*, if the word size doubles, *e* doubles too. Note that we are counting only logic and addition operations, not storage of values in program variables. Adding store operations would be more accurate but the number of these operations is compiler and optimization level specific.

We require that the alignment method be global or semi-global. That is, we do not restrict the initializations in the first row or column of the alignment scoring matrix or where in the last row or column the alignment score is obtained. Typical initializations require (i) a gap weight to be added successively to every cell (global alignment from the beginning of a sequence), and (ii) a zero in every cell (semi-global alignment where an initial gap has no penalty). We assume that match scores are positive or zero, M≥0, mismatch and gap scores are negative, I,G<0 and that the use of mismatch is possible, meaning that its penalty is no worse than the penalty for two adjacent gaps, one in each sequence, I≥2G. While other weightings are possible, they either reduce to simpler problems from a bit-parallel perspective (e.g. LCS has G=0,I=−∞,M=1) or require more complicated structures than detailed here (e.g. protein alignment using PAM or BLOSUM style amino acid substitution tables).

### 2.1 Function tables

Let *S* be a recursively-defined, global similarity scoring function for two sequences *X* and *Y* computed in an alignment scoring matrix:

$$S[i,j]=\max\{\begin{array}{cc} S[i-1,j-1]+M & \text{ if }\;X_{i}=Y_{j}\\ \;S[i-1,j-1]+I & \text{ if }\;X_{i}\neq Y_{j}\\ \;S[i-1,j]+G & \;\;\text{delete }\;X_{i}\\ \;S[i,j-1]+G & \;\text{ delete }\;Y_{j} \end{array}$$

Instead of actual values of *S*, we store only the differences, Δ*V*, between a cell and the cell above, and Δ*H*, between a cell and the cell to its left:

ΔV[i,j]=S[i,j]−S[i−1,j]

ΔH[i,j]=S[i,j]−S[i,j−1].

It is an easy exercise to prove that the minimum and maximum values for Δ*V* and Δ*H* are *G* and *M* − *G,* respectively. Lemma 2.1 gives the recursive definitions for Δ*V* and Δ*H* in terms of *M*, *I* and *G*.

**Lemma 2.1**

*The values for ΔV are as shown below and the values for ΔH are computed similarly. That is, *ΔH[i,j] *in matrix S is equal to *V[j,i] *in the transpose of matrix S.*

$$\begin{array}{l} \underset{\forall i,j\geq 1}{\Delta V[i,j]}=\{\begin{array}{cc} M-\Delta H[i-1,j] & Matc\mathrm{h,}\;i.e.\;:if\;X_{i}=Y_{j}\\ I-\Delta H[i-1,j] & Mismatch\text{, }\;i.e.\;:if\\ & I-G\geq \{\begin{array}{c} \Delta H[i-1,j]\\ \Delta V[i,j-1] \end{array}\\ G & Indel\;from\;above\text{, }\;i.e.\;:if\\ & \Delta H[i-1,j]\geq \{\begin{array}{c} I-G\\ \Delta V[i,j-1] \end{array}\\ \Delta V[i,j-1]+ & \\ G-\Delta H[i-1,j] & \;Indel\;from\;left\text{, }\;i.e.\;:if\\ & \Delta V[i,j-1]\geq \{\begin{array}{c} I-G\\ \Delta H[i-1,j] \end{array} \end{array}\\ \left(\underset{\forall j\geq 1}{V[0,j]}=G\;or\underset{\forall j\geq 1}{V[0,j]}=0\right) \end{array}$$

**Proof**

By substitution in the recursive formula for *S*. □

**Fig. 1.**
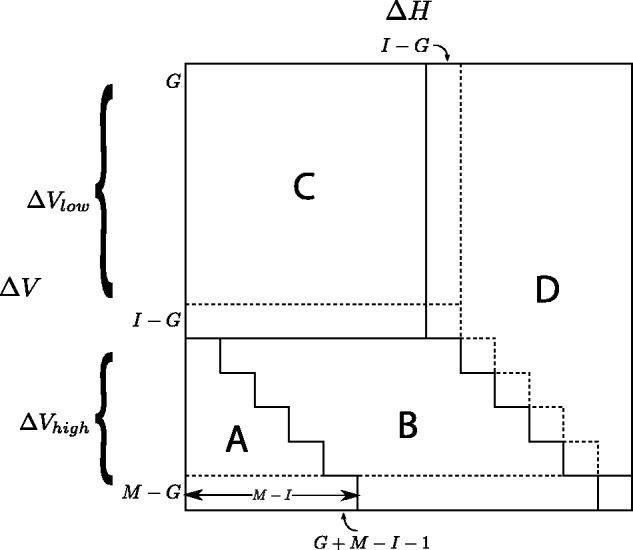
Zones in the Function Table for Δ*V*. Zone A: all values are in $V_{\text{high}}\in \{I-G+1,\ldots ,M-G\}$; Zone B: all values are in $V_{\text{low}}\in \{G,\ldots ,I-G\}$; Zone C: all values are in $V_{\text{low}}$ and values depend only on Δ*H*; Zone D: all values are G; Last row: values also apply when there is a match;. First column: identity column for values in $V_{\text{high}}$

**Fig. 2.**
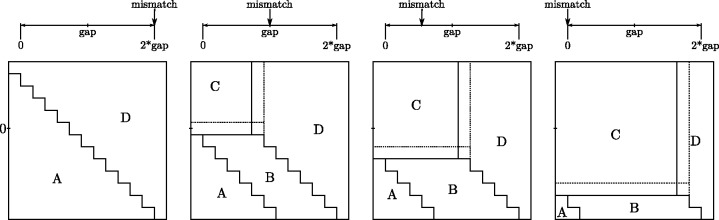
Relative size of Zones as *I* (mismatch penalty) decreases from 2*G* (twice gap penalty) where there is no preference for mismatches, to zero, where mismatches are free and gaps are introduced only to obtain matches

The recursion for Δ*V* is summarized in the Function Table in Figure 1. Note the value *I* − *G*, which frequently occurs in the recursion, and the relation ΔH=ΔV. They set the boundaries for the marked zones in the table. These zones comprise (ΔV,ΔH) pairs, which determine how the best score of a cell in *S* is obtained in the absence of a match, either as an indel from the left (Zones A and B), a mismatch (Zone C) or an indel from above (Zone D). Borders between zones, indicated by dotted lines, yield ties for the best score. Figure 2 shows how the relative size of the Zones changes with changes in *I* and *G*.

## 3 ALGORITHM

**Definitions**

min=G,max=M−G, mid=I−G, low ∈{min, …,mid} and high ∈{mid +1,…,max}.

For the illustrations in this article, we use the scoring weights:

M=2,I=−3,G=−5,

which yield

min=−5,max=7, mid=2,

low∈{−5,…,2},high∈{3,…,7}.

**Fig. 3.**
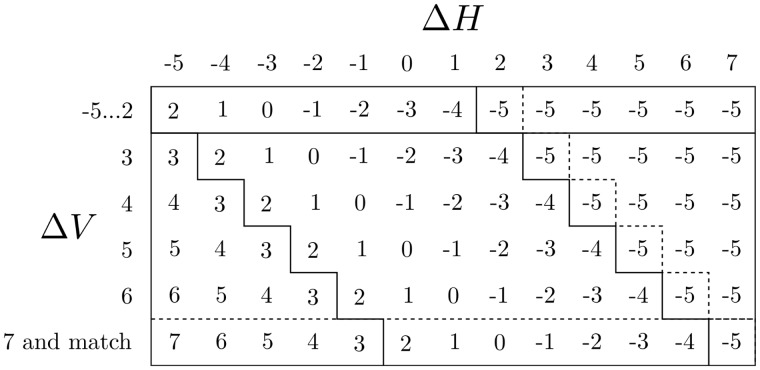
The Δ*V* Function Table for the weights M=2,I=−3,G=−5. Note that $\Delta V_{\text{high}},\Delta H_{\text{high}}\in \left[3,7\right]$; $\Delta V_{\text{low}},\Delta H_{\text{low}}\in \left[-5,2\right]$; $\Delta V_{\min}=\Delta H_{\min}=-5$; $\Delta V_{\max}=\Delta H_{\max}=7$. The Δ*H* Function Table is the transpose of this table, i.e. the labels Δ*H* and Δ*V* are swapped

The Δ*V* Function Table for these weights is shown in Figure 3.

The algorithm proceeds row-by-row through the alignment matrix. For each row, the input is:

- the Δ*H* values from the preceding row,
- the leftmost Δ*V* value in the current row and
- the match positions in the current row.

**Fig. 4.**
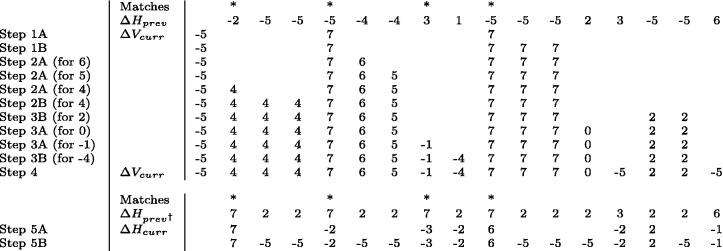
An example of the calculation of $\Delta V_{curr}$ and $\Delta H_{curr}$ values. $\Delta H_{prev}$ values come from the previous row. The match locations and the leftmost $\Delta V_{curr}$ value are known. The $\Delta V_{curr}$ value for a particular column is found using the table in Figure 3. The input is the $\Delta H_{prev}$ value in the same column and the $\Delta V_{curr}$ value in the column to the left, *except*, when there is a match, the value in the column to the left is treated as a max⁡ and, starting with Step 3, if the value in the column to the left is not assigned, it is treated as mid. $\Delta H_{prev}$^†^is a modification of $\Delta H_{prev}$ in which all Match positions have been changed to max⁡ and all values less than mid have been changed to mid. The $\Delta H_{curr}$ value for a particular column is found using the transpose of the table in Figure 3. The input is the $\Delta H_{prev}$^†^in the same column and the $\Delta V_{curr}$ value in the column to the left

The computation first determines all the remaining Δ*V* values for the current row and then, using those, determines the Δ*H* values for the current row. A central concept is a *run of* $\Delta H_{\min}$*.* This is a set of consecutive positions in the preceding row for which the values of Δ*H* all equal min (in Fig. 4, positions for which ΔH=−5).

The algorithm has the following steps (see Fig. 4), which follow from Lemma 2.1.

1. Find the locations where ΔV=max (highest value in Zone A):
**Step 1A:** because of a match between the characters in Sequence X and Sequence Y. These occur at match locations where ΔH=min.
**Step 1B:** in any run of $\Delta H_{\min}$ to the right of a match location in the run.
2. Find the locations where ΔV=i,fori∈{mid+1, …,max−1} (the remaining values in Zone A). These are computed in decreasing order of *i*. For each *i*, there are two categories, those locations:
**Step 2A:** because of a match or a larger preceding Δ*V* value. These also depend on the Δ*H* value.
**Step 2B:** because of the value *i* being carried through a run of $\Delta H_{\min.}$
3. Find the locations where ΔV=i, for i∈{min+1,…,mid} (the values in Zones B and C). These are computed separately for each value *i* and depend on:
**Step 3A:** a match or the preceding Δ*V* value and the Δ*H* value (Zone B).
**Step 3B:** the Δ*H* value alone (Zone C).
4. Find the locations where ΔV=min (the values in Zone D). These are:
**Step 4:** all the remaining locations with undetermined Δ*V* values.
5. Find the current row locations where the new ΔH=i for:
**Step 5A:** i>min.
**Step 5B:** i=min.

We describe the simplest case where the length of the first sequence is less than the computer word size *w*. Longer sequences can be handled in ‘chunks’, where each chunk has size *w*. Match positions for every row are computed before the calculation of the row values as is also done for the LCS and edit-distance problems. Details are given at the end.

We present two algorithms, **BitPAl** and **BitPAl Packed**. They differ in the data structures used to hold and process the Δ*H* and Δ*V* values and their computation of Steps 3, 4 and 5. Correctness theorems for the various steps are presented in Supplementary Information.

### 3.1 BitPAl

#### Data Structure for BitPAl

One computer word (sometimes called a vector) represents each possible value of Δ*H* and Δ*V*. Bit *i* in a word refers to column *i* in the alignment scoring matrix. With the weights used for illustration, there are 13 values {G,…,M−G}={−5,−4,…,6,7}, and therefore 13 words each, for Δ*H* and Δ*V*.

### Computing the Δ values

To compute its output values, each cell needs to know its Δ*H* and Δ*V* input values. As in standard left to right processing, the output Δ*V* value from one cell becomes the input value for the cell to its right. All the input Δ*H* values are in the preceding row.

#### Zone A

Inspection of the Function Table (Fig. 3) reveals that the output values in Zone A are interdependent and require computing in order from high to low. For example, output ΔV=5 can be obtained in two ways from higher Δ*V* input values, (ΔV=7,ΔH=−3) and (ΔV=6,ΔH=−4). ΔV=5 cannot be obtained from lower Δ*V* input values.

The leftmost column in the table, $\Delta H_{\min}$ (−5 in the example), is an identity column. This means that for runs of $\Delta H_{\min}$, an input Δ*V* value yields the identical Δ*V* ouput for every location in the run to the right of the input. For example, if the input ΔV=5 for the leftmost position in a run, then the output Δ*V* for every position in the run is also 5 (see Fig. 4 steps 1B, 2B for 4). Carrying an input value through a run of $\Delta H_{\min}$ can be accomplished with an addition (+) as seen below. Addition is similarly used to solve left-to-right dependency problems in LCS and edit-distance bit-parallel algorithms.

Note in the bottom row of the Function Table that a match acts as an input $\Delta V_{\max}$ (7 in the example), so we will treat the match positions as having input $\Delta V_{\max}$.

**Fig. 5.**
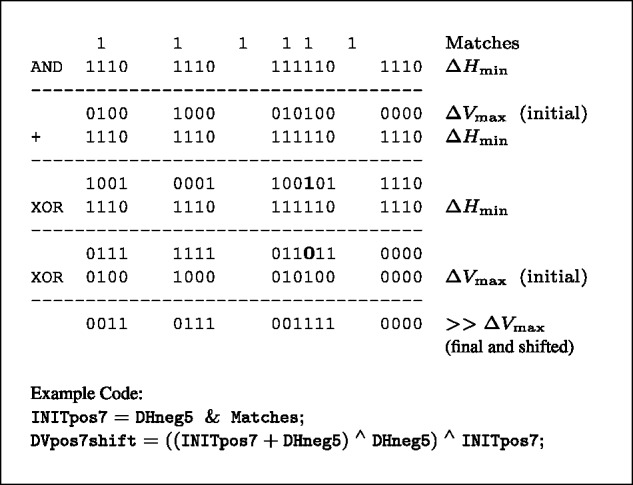
Finding $\Delta V_{\max}$. Each line represents a computer word with low order bit, corresponding to the first position in a sequence, on the left. 1s are shown explicitly, 0s are shown only to fill runs of $\Delta H_{\min}$ and the first position to the right of each run. Symbol >> indicates that the final $\Delta V_{\max}$ values are shifted to the right one position. Bits erroneously set by the ADDITION (+) are shown in bold. Sample code is from the complete listing in Supplementary Information

**Steps 1A and 1B:** The locations where ΔV=max, stored in the $\Delta V_{\max}$ vector, are calculated with four operations (Fig. 5). The locations are shifted one position to the right for input to subsequent calculations. The operations are—(i) an AND to find max because of matches; (ii) an ADDITION (+) to carry max through runs of $\Delta H_{\min}$ and into the position following a run (because the result will be shifted). This causes erroneous internal bit flips if there are multiple matches in the same run; (iii) an XOR with $\Delta H_{\min}$ to complement the bits within the $\Delta H_{\min}$ runs and (iv) an XOR with the initial $\Delta V_{\max}$ to correct any erroneous bits and finish the shift by removing the locations set with matches.

**Fig. 6.**
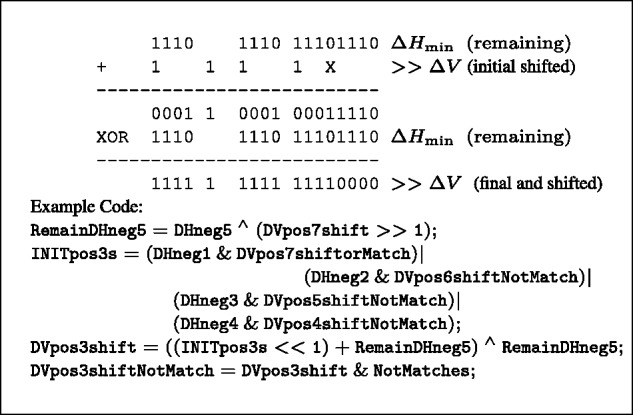
Carry through runs of $\Delta H_{\min}$ for remaining values in $\Delta V_{\text{high}}.$ Symbol X marks a single position between runs which cannot be 1 in the initial shifted values

**Steps 2A and 2B:** Remaining $\Delta V_{\text{high}}$ vectors are calculated, in descending order from ΔV=max−1 to ΔV=mid+1 because of the dependencies as discussed above. The operations are: (i) finding the locations because of a preceding higher Δ*V* value using AND of appropriate (ΔV,ΔH) pairs (which intersect along a common diagonal in the Function Table) and collecting them together with ORs; (ii) shifting the initial vectors right one position for subsequent calculations; (iii) carrying through runs of $\Delta H_{\min}$ computed in two operations, an ADDITION (+) as before and an XOR with $\Delta H_{\min}$ to complement the bits within the $\Delta H_{\min}$ runs (Fig. 6). Before the addition, those $\Delta H_{\min}$ positions that have already output a $\Delta V_{\max}$ value must be removed.

**Fig. 7.**
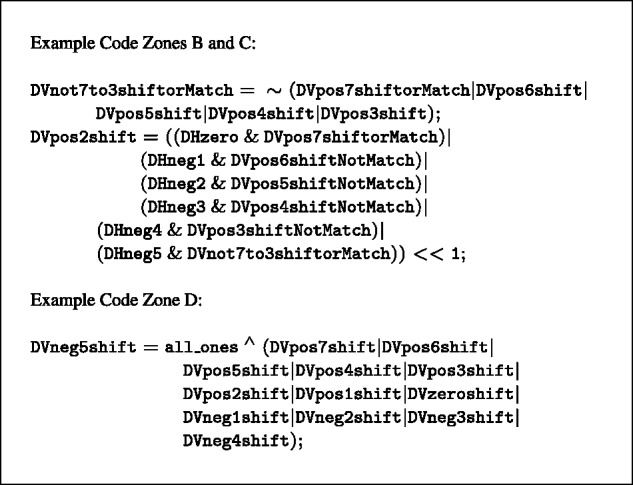
Code for Zones B, C and D

**Steps 3A and 3B.** (Fig. 7). At this point, all the $\Delta V_{\text{high}}$ input values for Zone B have been computed (they are the outputs from Zone A), remaining output values are all $\Delta V_{\text{low}}$. The operations are: (i) the AND of appropriate (ΔV,ΔH) pairs, which intersect along a common diagonal (Zone B); (ii) the AND of the appropriate Δ*H* vector and all positions without a $\Delta V_{\text{high}}$ output (Zone C); (iii) an OR combination of the preceding two results and (iv) a shift of the locations one position to the right for subsequent calculations.

**Step 4:** Zone D has only one output value, $\Delta V_{\text{min}}$. It is assigned to all remaining locations as well as the zero location if gap penalty in the first column is being used.

**Step 5:** After the Δ*V* values are computed, all inputs are available and the new Δ*H* vectors for the current row can be computed immediately. The Function Table for the new Δ*H* is the transpose of the table for Δ*V*, i.e. the input labels are swapped. Each new Δ*H* vector is obtained by the AND of appropriate (ΔV,ΔH) input pairs, which intersect along a common diagonal, collected together with ORs. Before this can proceed, though, the Match positions must be added to the previous row’s $\Delta H_{\max}$ vector (with OR) and removed from all other previous row Δ*H* vectors. Also, all previous row $\Delta H_{\text{low}}$ locations must be converted to $\Delta H_{\text{mid}}$.

### 3.2 BitPAl Packed

#### Data structure for BitPAl packed

The number of logic operations in BitPAl scales linearly with the size of the function table. Many of these are the AND and OR operations to compute identical values along Zone B diagonals. These calculations can be performed more efficiently with a new representation. The idea is to store the input Δ*H* and Δ*V* values in such a way that they can all be added simultaneously to give the appropriate output values.

**Fig. 8.**
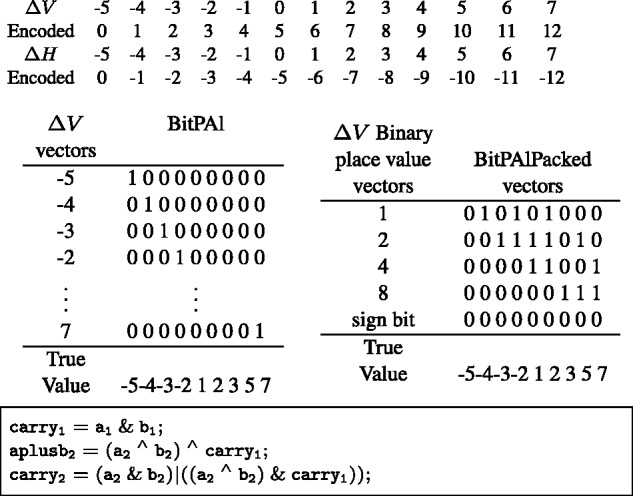
Top: The BitPAl Packed mapping of Δ*H* and Δ*V* values for the parameter set M=2,I=−3,G=−5. Middle: conversion from the 13 $\Delta V_{i}$ vectors at left to the five ‘packed’ vectors at right. Bottom: example code for adding the packed representation

Rather than using bit-vectors to represent single Δ*H* or Δ*V* values, we use them to represent binary digits (Fig. 8). We map the Δ*V* values {min,…,max} one-to-one onto the positive values {0,…,max−min} and store them in the vectors $\Delta V_{p0},\Delta V_{p1},\Delta V_{p2},$ etc. where *p_i_* is the place holder for the *i*th power of 2. The mapping for Δ*H* is onto negative numbers, i.e. {min,…,max} are mapped to {0,…,−(max−min)} and stored in vectors $\Delta H_{p0},\Delta H_{p1},\Delta H_{p2},$ etc. After addition, the sums will fall in {−(max−min),…,max−min}, so we use $\left\lceil \log_{2}(2(\max-\min)+1)\right\rceil$ bit-vectors for Δ*H* and Δ*V*. For our example, the Δ*V* values are mapped to {0,…,12}, the Δ*H* values are mapped to {0,…,−12} and the sums fall within {−12,…,12}, so we use five vectors each for Δ*H* and Δ*V*.

BitPAl Packed does not change the computation of the Δ*V* values in Zone A. The Δ*H* values are always maintained in the packed representation, but some are unpacked into the original representation for the Zone A computations. Once Steps 1 and 2 are completed, all locations without a Δ*V* value are set to mid, all match locations are set to max, and the Δ*V* values are converted into the packed representation.

Steps 3 and 4 are computed by ‘adding’ together the two sets of packed vectors using a series of AND, OR and XOR operations (Fig. 8) to produce the final encoded values for Δ*V*. Any negative values (sign bit set) are converted to min (Zone D). For Step 5, the new Δ*H* values are determined with a second addition. Because all input Δ*H* in the range [min,mid] give the same result, we first re-encode that range to mid.

#### Packing and unpacking

Packing Δ*V* vectors involves identifying the locations where the binary representation of the encoded values all have a specific bit set. For example, the binary representations for 1, 3, 5, 7, 9 and 11 all have the bit representing 2^0^ set, and the binary representations for 2, 3, 6, 7, 10 and 11 all have the bit representing 2^1^ set. Effectively then,

$$\Delta V_{p0}=\Delta V_{1}\text{ OR }\Delta V_{3}\text{ OR }\Delta V_{5}\text{ OR }\Delta V_{7}\text{ OR }\Delta V_{9}\text{ OR }\Delta V_{11}$$

$$\Delta V_{p1}=\Delta V_{2}\text{ OR }\Delta V_{3}\text{ OR }\Delta V_{6}\text{ OR }\Delta V_{7}\text{ OR }\Delta V_{10}\text{ OR }\Delta V_{11}$$

etc.

where $\Delta V_{i}$ is the vector of locations with encoded value *i*. However, as can be seen for these two examples, there are common terms ($\Delta V_{3},\Delta V_{7},\Delta V_{11}$), so combining the terms as above leads to inefficiencies.

Unpacking the Δ*H* vectors involves identifying locations of specific encoded values from the binary representation vectors. For example, the $\Delta H_{-1}$ locations are those (using two’s complement, −1 = 11 111) that have all bits set and $\Delta H_{-2}$ locations are those (using two’s complement, −2 = 11 110) that have all but the lowest bit set. Again, effectively

$$\Delta H_{-1}=\Delta H_{p0}\text{ \& }\Delta H_{p1}\text{ \& }\Delta H_{p2}\text{ \& }\Delta H_{p3}\text{ \& }\Delta H_{p4}$$

$$\Delta H_{-2}=∼\Delta H_{p0}\text{ \& }\Delta H_{p1}\text{ \& }\Delta H_{p2}\text{ \& }\Delta H_{p3}\text{ \& }\Delta H_{p4}$$

etc.

Again, there are common terms that can be combined to avoid inefficiencies. For both packing and unpacking, we use a binary tree structure in the code generator to guide creation of temporary intermediate vectors so that operations are not duplicated.

### 3.3 Other tasks

#### Determining matches

As a preprocessing step, the position of the matches are determined for each character *σ* in the sequence alphabet. A bit vector $Match_{\sigma }$ records those positions in sequence *X* where *σ* occurs. Filling all the $Match_{\sigma }$ simultaneously can be accomplished efficiently in a single pass through *X*.

#### Decoding the alignment score

The score in the last column of the last row of the alignment scoring matrix can be obtained by calculating the score in the zero column (=m*G) and then adding the number of 1 bits in each of the Δ*H* vectors multiplied by the value of the vector. Using the method described in (Kernighan and Ritchie, 1988), this takes O(n+M−2G) operations with a small constant:

$$S[m,n]=m*G+\sum_{i=G}^{M-G}{\text{bits}}_{i}*i$$

where ${\text{bits}}_{i}$ is the number of 1 bits set in $\Delta H_{i}$.

For BitPAl Packed, the alignment score can similarly be computed in O(n·k) operations

$$S[m,n]=m*G+\sum_{i=0}^{k-1}{\text{pbits}}_{i}*2^{i}.$$

where pbits_*i*_ is the number of 1 bits set in $\Delta H_{pi}$, and *k* is the number of bit vectors in the packed representation.

Several straightforward methods can be used to efficiently find all scores in the last row or last column.

### 3.4 Complexity and number of operations

The time complexity of our algorithms is O(znm/w) where *z* depends on the version. For BitPAl standard, *z* represents the combined size of Zones A, B and C (the latter reduced to a single row as in Fig. 3) in the Function Table. This in turn depends on the alignment weights *M*, *I* and *G*:

$$z=\frac{{\left(M-2G+1\right)}^{2}-{\left(I-2G\right)}^{2}}{2}$$

and the constant hidden in the big O notation is ∼4 (dominated by two operations per cell of Zones A, B and C for Δ*V* and separately for Δ*H*). For the example weights used in this article, the number of logic and addition operations, *p*, per word is 265, yielding an efficiency of 64/265≈0.24 cells per operation with 64 bit words.

For the packed version, *z* represents the size of Zone A, the number of distinct Δ*H* and Δ*V* values for the packing and unpacking steps, and the binary log of the number of distinct values for the addition steps:

$$z={\left(M-I\right)}^{2}+(M-2G+1)+\log_{2}(M-2G+1).$$

**Fig. 9.**
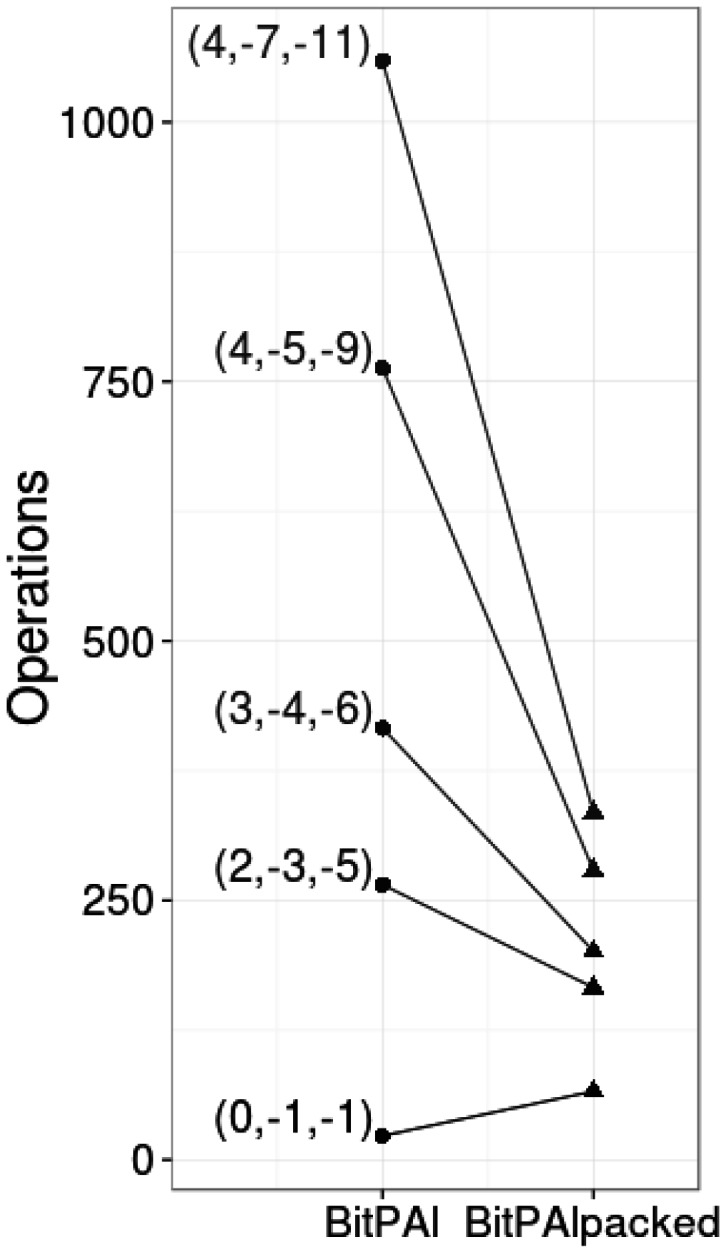
Comparison of the number of operations for BitPAl and BitPAl packed for different alignment weights (M, I, G)

Unlike the standard version, the term constants are not uniform (∼2, 2 and 12, respectively). For the example weights used in this article, the number of logic and addition operations, *p*, per word is 166, yielding an efficiency of 64/166≈0.38 cells per operation for 64 bit words. See Figure 9 for a comparison of the number of operations required by the two algorithms for different alignment weights.

### Implementation

Each unique set of weights *M*, *I* and *G* requires a uniquely tailored program. To simplify usage, we have constructed a Web site http://lobstah.bu.edu/BitPAl/BitPAl.html that generates C source code for download. The Web site takes as input the user’s alignment weights, the algorithm version (standard or packed), whether it will be used for short sequences (single word) or long sequences (multiple word) and where the final score should be found.

## 4 EXPERIMENTAL RESULTS

We compared running times for several bit-parallel algorithms using different alignment weights: (i) BitPal, (ii) BitPAl Packed, (iii) NW—the classical Needleman and Wunch (1970) dynamic programming alignment algorithm, (iv) LCS—the bit-parallel LCS algorithm of Hyyrö (2004), (v) ED—our improved bit-parallel, unit-cost edit-distance algorithm from the method of Hyyrö *et al.* (2005) and Myers (1999), (vi) WM—the unit-cost (Wu and Manber, 1992) approximate pattern matching algorithm and (vii) N—the (Navarro, 2004) general integer scoring, approximate regular expression matching algorithm. We implemented BitPAl, BitPAl Packed, NW, LCS, ED and WM. N was graciously provided by Gonzalo Navarro.

**Fig. 10.**
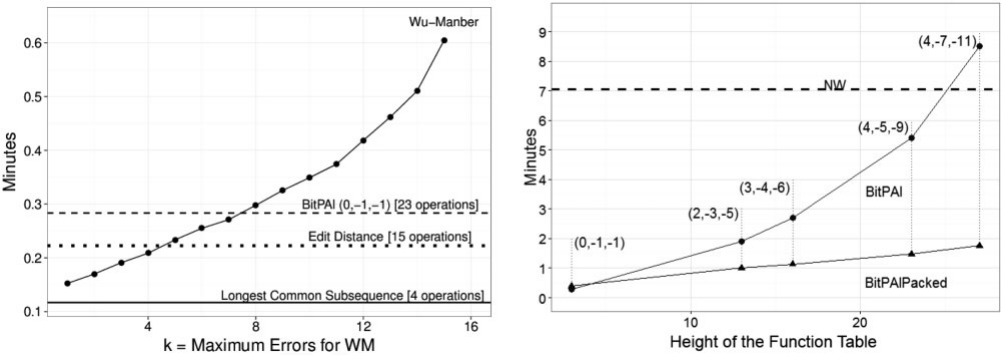
**Running times.** Each experiment involved 25 million alignments. For BitPAl and BitPAl Packed, alignment weights (M, I, G) are shown in parenthesis. All times are averages of three runs. **Left:** unit-cost BitPAl, unit-cost WM, LCS and ED. *k* is the maximum number of errors allowed for WM. *k* is not a parameter for the other algorithms and their times are shown as horizontal lines. LCS uses 4 bit operations per *w* cells, ED uses 15 bit operations, BitPAl (0, −1, −1) uses 23 bit operations. For *k* = 7, the times for BitPal and WM are nearly the same. By *k* = 15, BitPAl runs approximately twice as fast. Results for N are not shown on the graph. It was 118–304 times slower than BitPAl (0, −1, −1) even when optimal parameters were chosen. **Right:** variants of BitPAl and NW (shown as a horizontal line). For BitPAl, time is approximately linearly proportional to one dimension of the function table. For BitPAl packed, time is approximately linearly proportional to the area of the function tables. BitPAl packed (2, −3, −5) is ∼7.1 times faster than NW and BitPAl (0, −1, −1) is ∼24.9 times faster

For all experiments, we used human DNA and ran 100 pattern sequences against 250 000 text sequences for a total of 25 million alignments. (Pattern and text distinctions are irrelevant for BitPAl, BitPAl Packed, NW, LCS and ED.) All sequences were 63 characters long. For WM, we varied *k*, the maximum number of allowed errors, from 1 to 15. For N, we varied *k* from 1 to 12. All programs were compiled with GCC using optimization level O3 and were run on an Intel Core 2 Duo E8400 3.0 GHz CPU running Ubuntu Linux 12.10. Results are shown in Figure 10 and Table 1.

**Table 1.** Table of run times in minutes

Algorithm	Parameters (M, I, G)				
	0, −1, −1	2, −3, 5	3, −4, −6	4, −5, −9	4, −7, −11
BitPAl	0.284000	1.903778	2.702000	5.408722	8.517500
BitPAl Packed	0.390500	0.999945	1.126500	1.475222	1.755500

*Note*. Shown are averages over three trials for 25 million alignments. Needleman–Wunsch has the same runtime for all parameters, 7.056056 min.

## 5 DISCUSSION

The BitPAl and BitPAl packed algorithms outlined above can be extended in several ways. Computers now in common usage have special 128 bit SIMD registers (Single Instruction, Multiple Data). Using these, with the addition of several bookkeeping operations, would essentially double the efficiency and the speed of computation. Another extension derives from the unexploited parallelism of the operations. There are no dependencies on prior computations after the Δ*V* vectors in Zone A are computed. This means that all the computations in Zones B, C and D for Δ*V* and all the subsequent computations for Δ*H* can be done simultaneously, an ideal situation for the use of general purpose graphical processing units (GPGPU).

Another possible extension expands the types of scoring schemes allowed. BLOSUM type scoring, which is useful for protein alignments, eliminates match and mismatch scoring and instead assigns different substitution weights to each pair of characters. Affine-gap scoring replaces single character indel scoring with gap initiation and gap extension weights.

Extension to local alignment is also possible. This is a different class of problem in that the best final alignment score can occur in any cell of the alignment matrix. If all the cells have to be examined, then the time complexity shifts back to *O*(*nm*). Hyyrö and Navarro (2006) had some success with this problem using unit cost weights and identifying *columns* in which the score of at least one cell exceeds a predefined threshold *k*.

The BitPAl methods have already been used to accelerate software for detecting tandem repeat variants in high-throughput sequencing data (Gelfand *et al.*, 2014) and are well-suited to other DNA sequence comparison tasks that involve computing many alignments.

*Funding*: This work was supported by the National Science Foundation (IIS-1017621 to G.B., DGE-0654108 to J.L. and Y.H.).

*Conflict of interest*: none declared.

## Supplementary Material

Supplementary Data
