# Supplementary material for: BitPAl: a bit-parallel, general integer-scoring sequence alignment algorithm
Source: Bioinformatics. 2014 Jul 29;30(22):3166–73. doi: 10.1093/bioinformatics/btu507 (PMC4221118; doi:10.1093/bioinformatics/btu507)
Supplement: Supplementary Data [file supp_30_22_3166__index.html]

BitPAl: A Bit-Parallel, General Integer-Scoring Sequence Alignment Algorithm — BitPAl: a bit-parallel, general integer-scoring sequence alignment algorithm — BitPAl: a bit-parallel, general integer-scoring sequence alignment algorithm — Supplementary Data 

# BitPAl: a bit-parallel, general integer-scoring sequence alignment algorithm

## Supplementary Data

files

**Files in this Data Supplement:**

- Supplementary Data - pdf file
