# Supplementary material for: BitPAl: a bit-parallel, general integer-scoring sequence alignment algorithm
Source: Bioinformatics. 2014 Jul 29;30(22):3166–73. doi: 10.1093/bioinformatics/btu507 (PMC4221118; doi:10.1093/bioinformatics/btu507)
Supplement: Supplementary Data [file supp_btu507_appendix.pdf]

APPENDIX

Definitions
$Max = M - G$, the largest possible value for ΔV or ΔH.
$Min = G$, the smallest possible value for ΔV or ΔH.
$Mid = I - G$, the value that marks the border between Zones A and B and Zone C.
ΔV_i and ΔH_i: bit-vectors that represent the locations of the ΔV or ΔH value $i \in [Min, Max]$.
$<< 1$: a shift of one bit toward the higher order bits in a bit-vector, with the insertion of a 0 at the lowest order bit.
$\Delta V_i^{<<}$: notation for $\Delta V_i << 1$. The shift prepares the output of one cell for input to the next.
Matches: a bit-vector representing the locations of the matches.
Block in ΔH_{min}: within ΔH_{min}, a region in which there are several contiguous bits set to the same value (either 0 or 1).

The following theorems refer to Figure 1 which shows the relationship between values in four adjacent cells of an alignment scoring matrix.

$$\Delta V \left\{ \overbrace{\begin{array}{ccc} x & \xrightarrow{h} & y \\ v\downarrow & & \downarrow u \\ w & \rightarrow & z \end{array}}^{\Delta H} \right.$$

Fig. 1. The relationships between scores in adjacent cells in the scoring matrix: w, x, y, z are scores, h, v, u are differences: $h = y - x$, $v = w - x$, $u = z - y$.

Function Table. Theorem 6.1 defines the function table for ΔV. The function table for ΔH is identical but transposed.

THEOREM 6.1. *Given x, y, w and z as in Figure 1, match score $M \geq 0$, mismatch score $I < 0$ and gap (indel) score $G < 0$, ΔV input v and ΔH input h, with $v, h \in \{Min, Min+1, \ldots, Max\}$, the output ΔV value u is:*

$$u = \begin{cases} M - h, \text{ if there is a match, for } h \in \{Min, \ldots, Max\} \\ \qquad \text{(Match case) (1)} \\ I - h, \text{ if } v, h \in \{Min, \ldots, Mid\} \text{ (Zone C) (2)} \\ v - h + G, \text{ if } v \in \{Mid+1, \ldots, Max\} \text{ and } v > h \\ \qquad \text{(Zones A and B) (3)} \\ G, \text{ otherwise (Zone D) (4)} \end{cases}$$

PROOF. From the similarity recurrence formula:

$$z = \max \begin{cases} x + M & \text{if match} \\ x + I & \text{if mismatch} \\ w + G & \text{horizontal gap} \\ y + G & \text{vertical gap} \end{cases}$$

Match case: Suppose that there is a match. Then

$$\begin{aligned} z &= \max(x + M, w + G, y + G) \\ &= \max(x + M, x + v + G, x + h + G) \end{aligned}$$

but, $v, h \leq M - G$. Taking the largest value creates equality in all three terms, so $z = x + M$ for all values of v, h. Substituting,

$$u = z - y = z - (x + h) = z - x - h = M - h.$$

Mismatch case (Zone C): Suppose that there is a mismatch and $z = x + I$. Then

$$x + I \geq w + G \geq x + v + G \Rightarrow Mid = I - G \geq v$$
$$x + I \geq y + G \geq x + h + G \Rightarrow Mid = I - G \geq h$$

so $h, v \in \{Min, \ldots, Mid\}$. Substituting,

$$u = z - x - h = I - h.$$

Horizontal gap (Zones A and B): Suppose z comes from a horizontal gap only. Then $z = w + G$ and

$$w + G > x + I \Rightarrow x + v + G > x + I \Rightarrow v > I - G = Mid$$
$$w + G > y + G \Rightarrow x + v + G > x + h + G \Rightarrow v > h.$$

Then $v \in \{Mid + 1, \ldots, Max\}$ and $v > h$, case (3). Substituting,

$$u = z - x - h = w + G - x - h = w - x - h + G = v - h + G.$$

Vertical gap (Zone D): Suppose z comes from a vertical gap. Then $z = y + G$ and

$$y + G \geq w + G \Rightarrow x + h \geq x + v \Rightarrow h \geq v$$
$$y + G \geq x + I \Rightarrow x + h + G \geq x + I \Rightarrow h \geq I - G = Mid.$$

Since $z = y + G$, $u = G$. □

Output ΔV values. Theorems 6.2–6.5 are used to compute the ΔV output values for the four zones of the function table. The proof for ΔH values is omitted.

Zone A.

THEOREM 6.2. *(Zone A max value.) Given the bit-vector ΔH_{min} and the bit-vector Matches, the bit-vector $\Delta V_{max}^{<<}$ ($\Delta V_{max} << 1$) can be computed using the following equation:*

$$\begin{aligned} &\Delta V_{max}^{<<} = \\ &\quad (((\Delta H_{min} \wedge Matches) + \Delta H_{min}) \oplus \Delta H_{min}) \\ &\quad \oplus (\Delta H_{min} \wedge Matches) \end{aligned}$$

PROOF. Let *left* be the direction of the least significant bit and *right* the direction of the most significant bit. There are two ways for u to equal Max, either $h = Min$ and there is a Match or $h = Min$ and $v = Max$. Let

$$InitialV_{\text{max}} = \Delta H_{\text{min}} \wedge Matches.$$

Then $InitialV_{\text{max}}$ represents all the positions where $u = Max$ because of a Match. We consider two cases: a block of consecutive 1s in ΔH_{min} and a block of consecutive 0s.

Block of consecutive 1s: Let *Matches* contain k 1s at locations $\{p_1, p_2, \ldots, p_k\}$ within the block with p_1 the leftmost at the dth location within the block. $InitialV_{\max}$ also has 1s at these locations and nowhere else in the block. The operation $InitialV_{\max} + \Delta H_{\min}$ adds these 1s and causes a carry from p_1 to the end of the block. In the result, 1s occupy all positions left of p_1 in the block, positions $\{p_2, p_3, \ldots, p_k\}$, and the position immediately to the right of the block, if it exists. When we XOR this result with $\Delta H_{\min}$, the 1s left of p_1 are set to 0, p_1 is set to 1, the 0s between the p_is are set to 1s, and the positions $\{p_2, p_3, \ldots, p_k\}$ are set to 0. The bit to the right of the block remains 1. The final XOR of the result and $InitialV_{\max}$ sets p_1 to 0, and $\{p_2, p_3, \ldots, p_k\}$ to 1, since in $InitialV_{\max}$ those positions are all 1. The final result is a block of $n-d+1$ 1s, starting at position $p_1 + 1$ and ending at the first position to the right of the block.

Block of consecutive 0s: Within the block, $InitialV_{\max}$ is all 0s because $\Delta H_{\min}$ was 0. Likewise $InitialV_{\max} + \Delta H_{\min}$ is all 0s, unless a carry has entered the block from the left, in which case the leftmost bit in the region is a 1. When we XOR this result with $\Delta H_{\min}$, the output is again all 0s, aside from the possible initial 1 bit. After the final XOR with $InitialV_{\max}$ the output is again all 0s, except possibly the initial bit.

Bits set to 1 now occupy all locations where $u = Max$, *i.e.*, either from $h = Min$ and there is a Match, or $h = Min$ and $v = Max$, all shifted one bit to the right. □

THEOREM 6.3. *(Zone A Remaining Values.) Let* $u \in \{Max - 1, Max - 2, \ldots, Mid + 1\}$ *be a* ΔV *output value in Zone A. Then the output bit-vector* $\Delta V_u^{<<}$ *can be computed by the equation*

$$\Delta V_u^{<<} =$$
$$[(\Delta H_{M-u} \wedge Matches) \vee \quad (1)$$
$$(\bigvee_{\substack{k,l | l-k+Min=u \\ k \neq Min \\ l > Mid}} (\Delta H_k \wedge (\Delta V_l^{<<} \wedge \neg Matches)))] << 1 \quad (2)$$
$$+ Remain\Delta H_{min} \oplus Remain\Delta H_{min} \quad (3)$$

where $Remain\Delta H_{min} = \Delta H_{min} \oplus (\Delta H_{min} \wedge Matches)$.

PROOF. From the function table, an output of u can be obtained in three ways: from a match, from ΔV input values $v \in \{Mid + 1, Mid + 1, \ldots, Max\}$, and from the propagation of u through a block of 1s in $\Delta H_{\min}$.

Formula Part 1: By Theorem 6.1 (1), when there is a match, $u = M - h \Rightarrow h = M - u$, so the bit-vector $\Delta H_{M-u} \wedge Matches$ gives locations where the output is u due to matches.

Formula Part 2: $(\Delta V_l^{<<} \wedge \neg Matches)$ excludes locations in $\Delta V_l^{<<}$ with $Matches$, since the value of ΔV is not used if there is a match. The ΔV_l values are shifted $(\Delta V_l^{<<})$ so that they are input to the next cell. Output values of u lie on the diagonal defined by ΔH_k and ΔV_l where $l - k + Min = u$. ANDing every such pair (excluding the pair where $k = Min$ and $l = u + 1$, which will be used in the last step) and ORing the result gives a bit-vector of the locations where the u output values come from the diagonal.

Parts 1 and 2 yield the locations in $\Delta V_u^{<<}$ that must be computed before propagating through blocks of $\Delta H_{\min}$.

The $<< 1$ operation shifts these output values one bit toward the high order bit so they can act as input to the next cell.

Formula Part 3: In Part 1 of Theorem 6.2, $\Delta H_{\min}$ inputs were used to produce u output. These locations must be excluded from the propagation, since they have already produced an output. $Remain\Delta H_{\min}$ is exactly $\Delta H_{\min}$ with all such locations excluded. The operations $+ Remain\Delta H_{\min} \oplus Remain\Delta H_{\min}$ carry out the propagation through the blocks of $\Delta H_{\min}$, as in the proof of theorem 6.2 and results in output values shifted by one bit to the right. □

Zones B and C.

THEOREM 6.4. *Let* $u \in \{Mid, Mid-1, \ldots Min+1\}$ *be a* ΔV *output value in Zones B or C.*

$$\Delta V_u^{<<} =$$
$$(\Delta H_{M-u} \wedge Matches) \vee \quad (1)$$
$$\left(\bigvee_{\substack{k,l | l-k+Min=u, \\ l > Mid}} [\Delta H_k \wedge (\Delta V_l^{<<} \wedge \neg Matches)] \right) \vee \quad (2)$$
$$\left[\Delta H_{I-u} \wedge \left(\neg \bigvee_{l=Max}^{Mid+1} \Delta V_l^{<<} \right) \right] \quad (3)$$

PROOF. From the function table, the ΔV output of u can be obtained in three ways: from a match, from ΔV input values $v \in \{Mid + 1, Mid + 2, \ldots, Max\}$ (Zone B), and from the ΔV input values $v \in \{Min, Min + 1, \ldots, Mid\}$ (Zone C) .

Formula Part 1: See proof of 6.3 Formula Part 1.

Formula Part 2: See proof of 6.3 Formula Part 2.

Formula Part 3: The ΔV values from Min to Mid have the same outputs in the function table, given the same ΔH input value. From Theorem 6.1 (2), since $v \in \{Min, Min + 1, \ldots, Mid\}$ and $u \neq Mid = G$, then $u = I - h$ and $h = I - u$. Since Zone A has already been computed, we know the ΔV values from Max to $Mid + 1$. Since the sets $\{Min, Min + 1, \ldots, Mid\}$ and $\{Mid + 1, Mid + 2, \ldots, Max\}$ are complementary, we find the locations of the ΔV values from Min to Mid by taking the bit-wise complement of the ORed bitvectors $\Delta V_{Mid+1}, \Delta V_{Mid+2}, \ldots, \Delta V_{\max}$. ANDing them to ΔH_{I-u} gives the locations of u. □

Zone D.

THEOREM 6.5. *(Zone D.) Suppose that the bit-vectors* $\Delta V_{max}^{<<}$, $\Delta V_{Max-1}^{<<}, \ldots \Delta V_{Min+1}^{<<}$ *have been computed (Zones A, B, and C). Then we can compute the bit-vector* $\Delta V_{min}^{<<}$ *with the equation:*

$$\Delta V_{min}^{<<} = \neg \left(\bigvee_{k=Min+1}^{max} \Delta V_k^{<<} \right).$$

PROOF. The locations of all previously computed ΔV outputs shifted 1 bit to the right is simply $\bigvee_{k=Min+1}^{max} \Delta V_k^{<<}$, so the locations that have Min output shifted 1 bit to the right must be $\neg \left(\bigvee_{k=Min+1}^{max} \Delta V_k^{<<} \right)$. □

BitPAl packed calculates the u values in Zones B and C by addition using an encoding which converts all ΔV values v into the following b values and all ΔH values h into the following c values:

$$b = v - Min$$
$$c = Min - h$$

The b values are zero or positive in the range $[0, Max - Min]$ and the c values are zero or negative in the range $[Min - Max, 0]$. The range of their sums is $[Min - Max, Max - Min]$.

Using individual bit-vectors to represent each ΔV or ΔH value results in very low information density - few bits are set compared to the overall number of bits. Instead, we use a twos complement encoding consisting of k bit vectors to store both the b and c encodings as above. The b values are stored in vectors $\Delta Vbits_{2^0}, \Delta Vbits_{2^1}, \ldots, \Delta Vbits_{2^k}$, the c values are stored in vectors $\Delta Hbits_{2^0}, \Delta Hbits_{2^1}, \ldots, \Delta Hbits_{2^k}$, and k is set to accommodate the range of sums, *i.e.*, $2^k \geq 2 * (Max - Min) + 1$ or $k = \lceil \log_2(2 * (Max - Min) + 1) \rceil$

For Zones B and C, all ΔV values $< Mid$ are treated as Mid. In this case we modify the encoding above so that

$$b = \begin{cases} Mid - Min & \text{if } v \leq Mid \\ v - Min & \text{otherwise} \end{cases}$$

The following theorem shows how to find output ΔV values using addition.

THEOREM 6.6. *(Packed Zones B and C.) Consider $v, h,$ and u from Figure 1 and let b and c be as in the encoding above. If $b + c > 0$, then $b + c = u - Min$, otherwise $u = Min$.*

PROOF.

$\boldsymbol{b + c > 0}$:
$\boldsymbol{v > Mid}$:
$b + c = v - Min + Min - h = v - h$ and $b + c > 0 \Rightarrow v - h > 0 \Rightarrow v > h$. From Theorem 6.1 (3), $u = v - h + G \Rightarrow u = b + c + G \Rightarrow b + c = u - G \Rightarrow b + c = u - Min$.
$\boldsymbol{v \leq Mid}$:
$b + c = Mid - Min + Min - h = Mid - h$ and $b + c > 0 \Rightarrow Mid - h > 0 \Rightarrow Mid > h$. From Theorem 6.1 (2), $u = I - h \Rightarrow u = Mid + Min - h \Rightarrow u - Min = Mid - h \Rightarrow b + c = u - Min$.
$\boldsymbol{b + c \leq 0}$:
$\boldsymbol{v > Mid}$:
$b + c = v - Min + Min - h = v - h$ and $b + c \leq 0 \Rightarrow v - h \leq 0 \Rightarrow v \leq h$. Then by Theorem 6.1 (4), $u = G = Min$
$\boldsymbol{v \leq Mid}$:
$b + c = Mid - Min + Min - h = Mid - h$ and $b + c \leq 0 \Rightarrow Mid - h \leq 0 \Rightarrow Mid \leq h$. Suppose that $Mid = h$. Then by Theorem 6.1 (2), $u = I - h = I - Mid = I - (I - G) = G = Min$. Suppose that $Mid < h$. Then by Theorem 6.1 (4), $u = G = Min$. □

Application to Distance Based Scoring

THEOREM 6.7. *For any distance based integer scoring scheme described by alignment weights (m, i, g) with $m = 0$, $i, g > 0$, $i \leq 2g$, the global alignment bit-parallel methods described above apply to an equivalent* similarity based *integer scoring scheme, with weights (M, I, G), with $M = 0$, $I = -i$, and $G = -g$.*

ΔV output (function table; columns: ΔH, rows: ΔV or match):

ΔV \ ΔH	I (+1)	S (0)	D (-1)
+1	0	+1	+1
0	0	+1	+1
-1 or match	-1	0	+1

ΔH output (function table; columns: ΔV, rows: ΔH or match):

ΔH \ ΔV	+1	0	-1
I	S	I	I
S	S	I	I
D or match	D	S	I

Fig. 2. Function tables for unit-cost edit-distance. ΔV output on left, ΔH output on right. I (Increase) $= +1$, S (Same) $= 0$, D (Decrease) $= -1$.

PROOF. Let $M = 0$, $I = -i$, and $G = -g$. Equivalence of the two scoring schemes for global alignment was established in (Smith and Waterman, 1981), Theorem 3, which states that for similarity scores M, I, G, the corresponding distance scores are $m = 0, i = M - I$, and $g = M/2 - G$. By substitution, $M, I,$ and G will produce $m, i,$ and g. □

The change in scoring weights is merely a remapping of the values for Max, Min, and Mid as defined above. The function table dimensions and Zones remain unchanged. Since $M \geq 0$, $k \geq 0$. Since $i, g > 0$, $i > 2k, g > k$.

EDIT-DISTANCE IN 15 OPERATIONS

Our best BitPAl algorithm for unit-cost edit-distance uses 23 operations. A faster bit-parallel algorithm was previously constructed by **?** as a simple modification of the k-differences, bit-parallel approximate pattern matching algorithm of Myers (1999) . The difference in the two algorithms amounts to a change in the values in column zero of the alignment scoring matrix. For pattern matching, there is no penalty for deletion of characters in the text before starting to match the pattern, so each cell in column zero has value zero. For global unit-cost edit-distance, deletion in each string from the beginning is penalized, so the value in cell i of column zero is i. Bit-parallel algorithms work on the difference in the values stored in adjacent cells, and the difference, for edit-distance, between column zero cells in row i and $i - 1$ is always one. The modification made by **?** to the 15 operation algorithm of Myers (1999) was to add an additional operation to put the one in the initial bit of the bit-vector representing the differences between vertically adjacent cells in a row. That modification has 16 operations. Below we show how to achieve global, unit-cost edit-distance in 15 operations.

The algorithm assumes the ΔH values for the previous row are available. Three differences are possible, $-1, 0,$ and $+1$, and in what follows, these are designated D (decrease), S (same), and I (increase). Since there are only three values, it is enough to save vectors for two and derive the third if necessary. The **key** to this algorithm is that the two vectors that we save are D and $S|D$, the union of S and D. They contain enough information to derive S and I, but in our method these are not needed explicitly. However, $S|D$ is used three times and having it in hand saves one operation.

Several observations, derived from the function table (Figure 2), form the basis of our approach:

1. D always gives the same output, $\Delta V = +1$, whether there is a match or not, so it can always be treated as a match. To represent this, we use a vector M_m which is the union of the Match and D positions
2. ΔH runs of *non-match* I or *non-match* S are central to the algorithm. We represent these in the vector $R_{I|S}$, which is the union of all non-match I positions and non-match S positions. Note that $R_{I|S}$ is the complement of M_m.

   a. In runs preceded by $\Delta V = 0$ or $+1$, all I output 0, all S output +1. These runs are designated $_{+1|0}R_{I|S}$.

   b. In runs preceded by $\Delta V = -1$, all I left of the first (leftmost) S in the run output -1, all other I ouput 0. The first S outputs 0, all other S ouput +1. These runs are designated $_{-1}R_{I|S}$.

The central steps of the algorithm are shown in Figure 4. Below is a description of steps.

- Steps 1 – 3 set up vectors which are used more than once: M_m, $R_{I|S}$, and $notM_I$, which is all non-match I.
- Step 4 is shown separately for clarity. It computes $R_{I|S}orS$, *i.e.*, a vector which includes the $R_{I|S}$ runs and all match S outside the runs. The computation is through a backdoor, so that S does not need to be computed explicitly.
- Steps 5 and 6. The ADDITION in Step 5 is set up to force a carry of 1 into every $R_{I|S}$ run preceded by S or D ($_{+1|0}R_{I|S}$) and no carry into every run preceded by a match I ($_{-1}R_{I|S}$). In the case of the forced initial carry, every position in the run produces a carry. All I positions are set to 0 and all S positions are set to 1. In the case of no initial carry, the first (leftmost) S causes the first carry. All I to the left of the first S are set to 1 and all I right of the first S are set to 0. The first S is set to 0 and all remaining S are set to 1. The AND in Step 6 is used to mask out all but the results in the $R_{I|S}$ runs. All other positions are set to 0.
- Step 7 defines ΔV_0, the vector for positions where $\Delta V = 0$. The second term in the XOR contains a 1 at each $R_{I|S}$ position and each match S position. It therefore complements the results inside the $R_{I|S}$ runs from Step 6 and puts a 1 in every match S position outside the runs. For $_{-1}R_{I|S}$ runs, the first S and all I to its right are set to 1 and for $_{+1|0}R_{I|S}$ runs, all I are set to 1. Inspection of the function table confirms that these are correctly set. Output $\Delta V = 0$ occurs at

  1. match S. This is all S outside the $R_{I|S}$ runs.
  2. S and input $\Delta V = -1$. This is the first S in $_{-1}R_{I|S}$ runs.
  3. non-match I and input $\Delta V \neq -1$. These are the I in $_{+1|0}R_{I|S}$ runs and the I in $_{-1}R_{I|S}$ runs that follow the first S.

- Step 8 defines ΔV_{+1}, the vector for positions where $\Delta V = +1$. The first term puts a 1 in every D position. The AND in the second term selects the S positions in the $R_{I|S}$ runs that were set to 1 in Step 6 (all S except the first ones in $_{-1}R_{I|S}$ runs). Again, inspection of the function table shows that these are correctly set. Note that only S is required for the AND, but $S|D$ is available and works as well.

Step

****** Setup ******

1. $M_m = M \mid D$
2. $R_{I|S} = \sim M_m$
3. $notM_I = R_{IS} \mid S|D$
4. $R_{I|S}orS = notM_I \wedge D$

****** the add ******

5. Sum $= notM_I + S|D$
6. MaskSum = Sum & $R_{I|S}$

****** new ΔV vectors ******

7. $V_0 =$ MaskSum $^\wedge$ $R_{I|S}orS$
8. $V_{+1} = D \mid$ (MaskSum & $S|D$)

****** new ΔV vector shifts ******

9. $V_0^{<<} = (V_0 << 1)$
10. $V_{+1}^{<<} = (V_{+1} << 1)$

****** penalty for column zero ******

11. $V_{+1}^{<<} += 0x0000000000000001$

****** new ΔH vectors ******

12. $D = M_m$ & $V_{+1}^{<<}$
13. $S|D = V_{+1}^{<<} \mid (M_m$ & $V_0^{<<})$

Fig. 4. 15 Operation unit-cost edit-distance algorithm

- Steps 9 and 10 shift the ΔV vectors 1 bit so they can be used as input to compute the new ΔH vectors.
- Step 11 sets the bit in the first column of the ΔV_{+1} vector so that global edit-distance is computed.
- Step 12 sets the new D. From the function table, output $\Delta H = D$ when input ΔV is +1 and 1) there is a match or 2) input ΔH is D. M_m stores the latter two conditions.
- Step 13 sets the new $S|D$. From the function table, output $\Delta H = S$ or D when ΔV is +1 (first column of table), or $\Delta V = 0$ and 1) input $\Delta H = D$ or 2) there is a match. Again, M_m stores the latter two conditions.

REFERENCES

Hyyrö, H. and Navarro, G. (2005). Bit-parallel witnesses and their applications to approximate string matching. *Algorithmica*, **41**(3), 203–231.

Myers, G. (1999). A fast bit-vector algorithm for approximate string matching based on dynamic programming. *Journal of the ACM (JACM)*, **46**(3), 395–415.

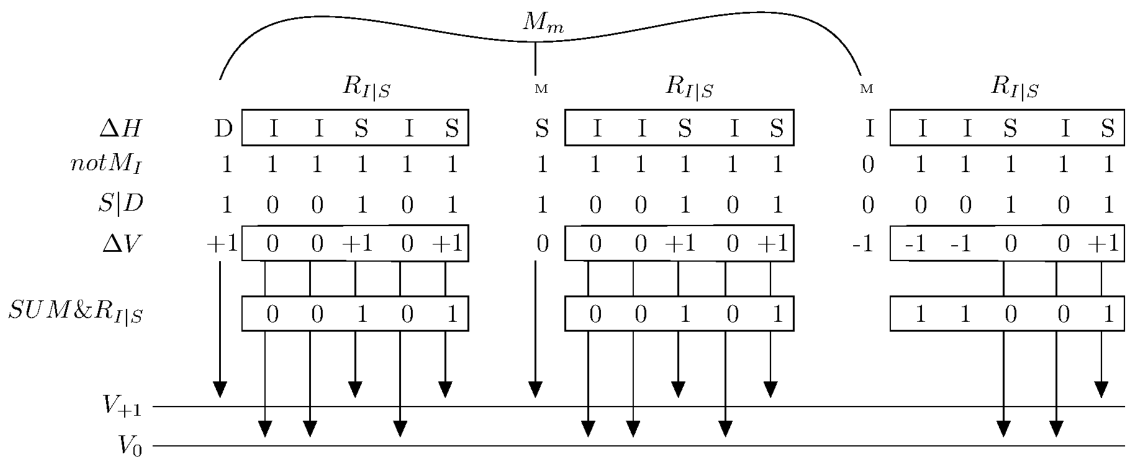

Fig. 3. Illustration of steps 1–8. M_m consists of all matches and all D. $R_{I|S}$, shown in boxes, consists of all non-match I or S. $notM_I$ is everything but match I. Adding $notM_I$ and $S|D$ and masking with AND of $R_{I|S}$ produces $MaskSum$, lower boxes. V_{+1} comes from any D or any S set to 1 in $MaskSum$. V_0 comes from any match S or any 0 in $MaskSum$.
